# Supplementary figures and images for: Long-term changes in populations of rainforest birds in the Australia Wet Tropics bioregion: A climate-driven biodiversity emergency
Source: PLoS One. 2021 Dec 22;16(12):e0254307. doi: 10.1371/journal.pone.0254307 (PMC8694438; doi:10.1371/journal.pone.0254307)

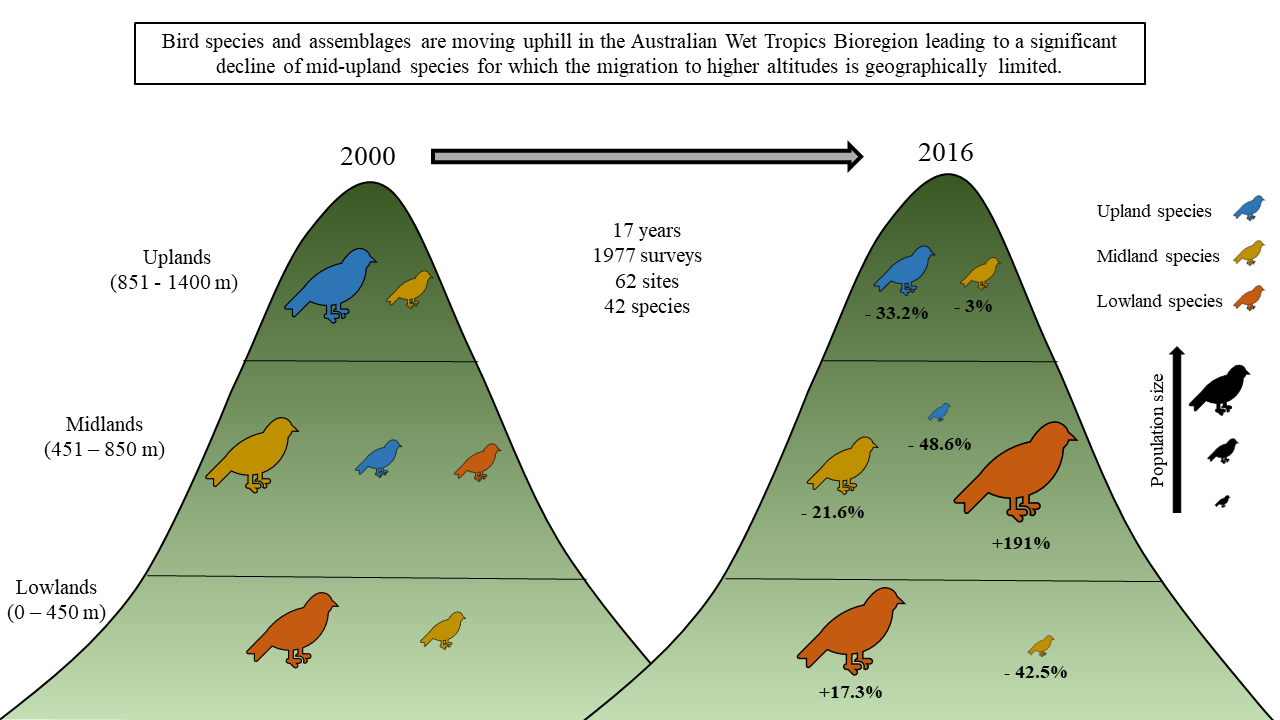

Supplement: S1 Graphical Abstract — (PNG) [file pone.0254307.s002.png]
